# Advances in the Study of Metabolomics and Metabolites in Some Species Interactions

**DOI:** 10.3390/molecules26113311

**Published:** 2021-05-31

**Authors:** Rui Liu, Zheng-Xue Bao, Pei-Ji Zhao, Guo-Hong Li

**Affiliations:** State Key Laboratory for Conservation and Utilization of Bio-Resources in Yunnan, and Key Laboratory for Microbial Resources of the Ministry of Education, Yunnan University, Kunming 650091, China; ruiliu991@outlook.com (R.L.); bzx357489@126.com (Z.-X.B.); pjzhao@ynu.edu.cn (P.-J.Z.)

**Keywords:** interactions, metabolomics, metabolites, species, analysis technologies

## Abstract

In the natural environment, interactions between species are a common natural phenomena. The mechanisms of interaction between different species are mainly studied using genomic, transcriptomic, proteomic, and metabolomic techniques. Metabolomics is a crucial part of system biology and is based on precision instrument analysis. In the last decade, the emerging field of metabolomics has received extensive attention. Metabolomics not only provides a qualitative and quantitative method for studying the mechanisms of interactions between different species, but also helps clarify the mechanisms of defense between the host and pathogen, and to explore new metabolites with various biological activities. This review focuses on the methods and progress of interspecies metabolomics. Additionally, the prospects and challenges of interspecies metabolomics are discussed.

## 1. Introduction

Metabolites are the core of the interrelation between cell changes and phenotypes, which directly reflect the physiological state of cells [1,2]. Metabolomics is the branch of science concerned with the qualitative and quantitative analysis of the metabolites of integrated living systems and their dynamic responses to changes in the environment [3,4,5], and the detection, analysis, and identification of metabolites are the core of metabolomics. The advances in chromatography coupled with mass spectrometry have allowed scanning almost all polar and semi-polar metabolites of low molecular weight (typically 50~1000 Da) in an organism at once [6], such as gas chromatography–mass spectrometry (GC-MS), liquid chromatography-mass spectrometry (LC-MS), and nuclear magnetic resonance (NMR), which are the most commonly used methods. These metabolomics methods have developed rapidly over the past decade, playing a crucial role in analyzing metabolic pathways, identifying microbial types, researching gene functions and transcriptome and proteome associations, and providing new methods and ideas for research. There have been many excellent reviews on metabolomics in recent years [7,8,9].

The application of metabolomics in analyzing the interactions between different species not only provides a broad picture of metabolic pathways, but also explains the mechanisms of microbial and host interactions. Based on metabolomics, a comprehensive analysis of interactions can effectively identify the functions of the genes involved in defense mechanisms. Combined with genetic means, metabolomics can play a role in screening resistant varieties and auxiliary breeding in practice. In this paper, we mainly summarize the advantages and disadvantages of each metabolomics analysis platform and its application in the interaction of different species. This not only helps in understanding to elucidate the application of metabolomics in the pathogenic and defense mechanisms of pathogens, but should also help increase the biotechnology development, specifically metabolomics strategies.

## 2. Data Sources

Publications were found from the Web of Science and PubMed. “Metabolomics,” “Interactions,” and “Analysis technology” were used as the keywords for the literature search. The document types were journals and reviews. The retrieval time coverage was from January 2000 to May 2020.

## 3. Analysis Technologies for Metabolomics

The detection, analysis, and identification of metabolites are the core of metabolomics. Although metabolomics research has developed rapidly in recent years, nuclear magnetic resonance (NMR) and mass spectrometry (MS) are the two main measurement techniques. NMR can provide excellent reproducibility and quantitative accuracy, as well as unambiguous data to elucidate compound structures via nondestructive measurement [10]. Meanwhile, MS and MS^n^ can provide informative and accurate mass determination, structural identification, and can assessable contents at high sensitivities. Furthermore, MS instrumentation can also be accessible on the level of the analytical resolution at a low price. For these reasons, MS has become the most prevalent metabolomics technique. In general, MS or NMR systems couple into separation devices. Separation technologies such as liquid chromatography (LC), gas chromatography (GC), and capillary electrophoresis (CE) improve metabolite identification and quantitation by reducing the complexity of samples. Recently, there have been significant advances in separation and MS system integration interfaces, chromatographic separation efficiency, MS resolution, detector sensitivity, and data acquisition [11,12].

Below, we list several common technologies used in metabolomics and summarize their advantages, disadvantages, and applicability.

### 3.1. Gas Chromatography-Mass Spectrometry

Gas chromatography–mass spectrometry (GC-MS) is an early and frequently used method in metabolomics platforms. GC-MS has the advantages of good separation performance, easy operation, and being more economical. It can simultaneously analyze hundreds of compounds, and is equipped with a standard metabolite spectrum library, which can quickly and accurately qualitatively analyze metabolites. However, GC-MS has obvious drawbacks: Although it is suitable for volatile and non-thermosensitive compounds, GC-MS is not as suitable for less volatile compounds, and GC-MS may change in some compounds due to the effort required for derivatives. In addition, although high-resolution data only slightly improve conventional library matching, with its clear benefits in terms of sensitivity and metabolic coverage, high-resolution gas chromatography (HRGC) is still the most suitable technique for the analysis of volatile compounds [13]. At present, GC–MS is mostly used in targeted metabolomics studies or as a supplement to non-targeted metabolomics LC–MS [14,15].

The multi-dimensional separation equipment (such as two-dimensional gas chromatography coupled with time-of-flight mass spectrometry (GC×GC-TOF-MS) developed in recent years will have more advantages in the future due to its strong specificity and high sensitivity [16]. GC×GC has the characteristics of high resolution, large peak capacity, high sensitivity, and short analysis time [17]. Therefore, in series with TOF, GC×GC-TOF-MS can not only accurately separate metabolites from samples, but can also accurately determine the relative molecular weight of metabolites. Using GC×GC-TOF-MS, Beckstrom detected the plasma metabolites of six non-human primates before and a few days after delivery, and found that 100 metabolites changed during delivery [18,19].

Compared with one-dimensional GC, the pairing of comprehensive two-dimensional gas chromatography (GC × GC) and high-resolution mass spectrometry (HRMS) provides higher peak capacities for target analysis.

### 3.2. Liquid Chromatography–Mass Spectrometry

Similar to GC-MS, liquid chromatography-mass spectrometry (LC-MS) is also applied in metabolomics to analyze and detect compounds, but it makes up for the shortcomings of GC-MS. LC-MS is suitable for metabolites with low volatility and poor thermal stability and is another important platform in metabolomics research [20,21,22]. Thus, an analysis method based on LC-MS is convenient for metabolomics analysis samples, especially by reversed-phase (RP) separation technology. Parts of the samples can be directly injected into the chromatographic column without any pretreatment [23]. This reversed-phase gradient elution separation is suitable for the analysis of middle- and low-polar compounds, whereas more polar compounds (such as amino acids and sugars) can be characterized by hydrophilic exchange chromatography (HILIC) [24].

At present, the main problems faced by pharmacokinetic research are the large amount of test samples, difficult separation, the large amount of matrix interference components, and the low content of samples. Due to the advantages of high sensitivity, high selectivity, high-throughput and detectability of residual compounds, liquid chromatography-tandem mass spectrometry (LC-MS/MS) can not only avoid complex, tedious, and time-consuming sample processing work, but it can separate and identify the trace drug metabolites which are difficult to identify, can improve the specificity of analysis, and can improve the signal-to-noise ratio and sensitivity through multi-reaction detection, so as to solve the above problems quickly and conveniently. Jeml et al. reviewed the application of LC-MS/MS in the detection of drug prototypes and metabolites in blood and urine in a drug research in recent years. This method has brought about rapid and highly sensitive quantitative bioanalysis [25].

However, due to the large amount of interference components in the biological sample matrix, co-eluting substances affect the ionization of the analyte and increase or decrease the mass spectrum response of the analyte, thus affecting the accuracy and precision of the LC-MS/MS analysis method. The matrix effect is a special problem in LC-MS/MS analysis, so it is very important to overcome or reduce this effect.

Although LC-MS has been applied in many studies, there are still some problems in metabolomics. For example, a high salt concentration in the medium inhibits the ionization efficiency of ESI and affects the efficiency and repeatability of quantitative analysis.

### 3.3. Capillary Electrophoresis-Mass Spectrometry

Capillary electrophoresis-mass spectrometry (CE-MS) is a good integration of modern micro-column separation and electrophoresis technologies to achieve efficient, micro-, and rapid separation of substances. CE-MS has developed rapidly in recent years. It has the characteristics of no special treatment for samples, low dosage, low reagent cost, fast analysis speed, high sensitivity, high-throughput, and short test time [26]. For strong polar metabolites that are easy to ionize, CE-MS can be used to obtain better analytical results compared to other technologies [27,28]. However, CE-MS has two main limitations: Sensitivity and reproducibility. The small amount of sample injected is a drawback in terms of sensitivity, and then, time drift is a common problem in metabolic phenotyping.

### 3.4. Mass Spectrometry Imaging

A limitation of LC(GC/CE)-MS methods is the loss of spatial information resulting from metabolite extraction from homogenized samples. Thus, mass spectrometry imaging (MSI) is a potential tool for pathological analysis and the investigation of mechanisms, due to its capability to directly link molecular changes and histology. Sample processing for MSI is easy, in addition, MSI requires no fluorescence or radioisotope labeling, and has a wide mass range, enabling the analysis of elements, metabolites, peptides, and proteins. MSI is mainly classified according to the ionization mode (probe), which currently mainly includes the following three types: Secondary ion mass spectrometry (SIMS), which needs to be conducted in vacuum conditions [29], matrix-assisted laser desorption ionization (MALDI) [30], and desorption electrospray ionization (DESI) [31,32]. Although MSI is a new outstanding technology that enables us to determine the distribution of endogenous or exogenous molecules present in tissue, there are disadvantages in building an analytical platform for MSI: In MALDI-MSI, a new matrix development and its application is demanded. On the contrary, DESI-MSI has spatial resolution, and its value is approximately 200 μm, compared to 20 μm using MALDI. 

### 3.5. Nuclear Magnetic Resonance

Nuclear magnetic resonance (NMR) is a non-destructive, non-biased analysis technique that provides accurate information on the molecular structure of compounds. NMR has been widely used in the qualitative and quantitative analysis of metabolites. Proton NMR (^1^H-NMR) is extensively applied in metabolomics research for ^1^H atoms, which are present in most organic metabolites [33,34]. In addition to their omnipresence, ^1^H atoms exhibit the most NMR signal intensity with very high (~99%) isotopic natural abundance and show extraordinarily narrow line widths. Despite the many benefits of ^1^H-NMR spectra, there are several limitations to using NMR techniques. For a relatively narrow chemical shift range (of ~10 ppm), there is a greater likelihood of overlapping peaks in ^1^H-NMR spectra which leads to uncertainty in compound identification and quantification. In addition, ^13^C-NMR spectroscopy has a rather broad (~200 ppm) chemical shift range, but the application of ^13^C-NMR experiment is limited in metabolomics, due to the fact that the natural abundance of ^13^C (~1.1%) and the sensitivity of the ^13^C nucleus is low.

Spectral overlap can be efficiently reduced by increasing spectral dimensionality, and thus an increased interest has been shown in the use of two-dimensional (2D) NMR for quantitative analysis during the past decade. There are different homonuclear and heteronuclear 2D-NMR experiments, including ^1^H-^1^H correlation spectroscopy (COSY) and nuclear overhauser effect spectroscopy (NOESY) experiments along with ^1^H, ^13^C-heteronuclear single quantum correlation (HSQC), and heteronuclear multiple bond correlation (HMBC). The use of 2D-NMR for metabolomics has attracted attention during recent years, and target compounds are more easily demonstrated with 2D-NMR than with 1D-NMR [16,35]. However, since classic 2D-NMR spectra are acquired as a series of 1D spectra, the former take a longer time to acquire than the latter.

LC-NMR greatly improves the capabilities of NMR-based metabolomics. Combining LC with NMR takes advantage of the modern (HPLC and UPLC) chromatographic separation techniques to greatly reduce the complex sample. LC-NMR can be operated offline using fraction collectors and NMR tubes or inline using flow probes (being faster and simpler). LC-NMR has some disadvantages though. For example, large amounts of the solvent of a dilution sample reduce the sensitivity of sample detection during chromatographic separation [36,37].

In Table 1, we list the advantages and disadvantages of the above analysis technologies.

### 3.6. Structural Identification of Natural Products

The structural identification of natural products has a direct impact on the in-depth study of their biological activity and targeting effect, so the structural identification of compounds has always been regarded as one of the most critical and difficult works in the study of natural products. The quantity of natural products is huge and the structure type is various with, the determination of stereochemical structure being particularly difficult.

With the rapid development of science and technology, great changes have taken place in the research methods for the structures of natural products, and the dominant change is from the earliest chemical method instrumental analysis to spectrum analysis. Especially in the past 30 years, the application of modern spectral technology has greatly promoted the research of natural products [38].

In early studies, the structure determination of natural products mainly depended on chemical means, including a series of chemical reactions of functional groups, chemical degradation, derivative preparation, chemical conversion, and even total synthesis control. These methods are not only time- and labor-consuming, but also require a large amount of samples, and require researchers to have profound knowledge and skills in organic chemistry.

In the second half of the 20th century, due to MS and NMR, the combined technology of UV, IR, MS, and NMR in the structure identification of natural products became more and more mature, gradually replacing the chemical method, greatly accelerating structure identification. Since then, the four spectra have become routine in the laboratory, and structural identification is no longer a “daunting” task.

The application of traditional 1D- and 2D-NMR, X-ray diffraction, electron circle ECD, chemical degradation, and ICD can solve the structural identification problems of most natural products. However, traditional methods often encounter challenges when faced with structures, such as completely new skeleton molecules in liquid or colloidal states, flexible molecules, and polyheteroatoms or highly aromatic molecules. The development of quantum chemical calculation and computer technology provides new ideas for the successful solution of these difficult chemical problems.

Quantum chemical computation requires the skilled use of computers and specialized software. At present, the software that can run the quantum chemistry program include Gaussian, Spartan, ADF, Dalton, Gamess, Crystal, VASP, Wien, and DMOL.

## 4. Databases Linking Metabolomics

Many public databases store shared spectral information for metabolites and experimental profiles, including raw instrument files and annotated metabolite tables. In this paper, a list of the currently active databases that are commonly used for metabolomics is provided in terms of the content, data volume, and characteristics of each database.

The Golm Metabolome Database (GMD) is an open-access metabolomics database (http://gmd.mpimp-golm.mpg.de/ (accessed on 1 February 2020)) [39], which comprises custom mass spectral libraries, retention times, additional information and methods. In the current GMD, there are 26,590 total spectra. Of them, 2021 valid spectra are associated with metabolites and 11,680 are linked to the analyses.

PubChem is a very versatile, open-access database for small molecules (https://pubchem.ncbi.nlm.nih.gov/ (accessed on 1 February 2020)) [40]. PubChem provides the most complete publicly available repository of known compounds (containing 96,573,159), and is a very significant resource for annotating metabolomics data.

ChemSpider is a free, online chemical database (http://www.chemspider.com/ (accessed on 1 February 2020)), which contains the physical and chemical properties, chemical structure, spectral information, synthetic strategies, and nomenclature of almost 77 million compounds that are sourced and linked to almost 400 available data sources [41].

Global Natural Products Social Molecular Networking (GPNS) is a web-based mass spectrometry system (https://gnps.ucsd.edu/ (accessed on 1 February 2020)) [42], which intends to be an open access public mass spectrometry base for the community-wide organization and sharing of raw, handled or identified tandem mass (MS/MS) spectrometry data.

The METLIN Metabolite and Chemical Entity Database is a repository of experimental MS and tandem MS data acquired from standards (http://metlin.scripps.edu/index.php/ (accessed on 1 February 2020)) [43]. The tandem mass spectrometry information is provided to facilitate the identification of the chemical entities of over 450,000 compounds.

NMRShiftDB is an open-source, free-access, open-content public database for chemical structures and their NMR data (https://nmrshiftdb.nmr.uni-koeln.de/ (accessed on 1 February 2020)) [44]. This database contains 43,483 structures and assigned spectra. It provides (sub-) spectra and (sub-) structure searches and chemical shift prediction of ^13^C spectra on the basis of the current database information.

MassBank is a public repository for metabolomics and lipidomics research. It contains mass spectra from small compounds (http://massbank.jp/Index/ (accessed on 1 February 2020)) [45]. The database comprises 19,963 MS spectra and 36,505 MS/MS spectra, from different ion sources such as APCI, ESI, EI, FAB, FD, FI, APPI, and MALDI.

Massbank of North America (MoNA; https://mona.fiehnlab.ucdavis.edu/ (accessed on 1 February 2020)) is an important MS database for the identification of metabolites. At present, this database contains 74,833 metabolites and 212,000 spectra, covering a variety of different mass spectrograms together. These data come from several available spectrogram libraries, including experimental databases (such as MassBank and HMDB) and theoretical databases (LipidBlast).

The Human Metabolome Database (HMDB; http://www.hmdb.ca/ (accessed on 1 February 2020)) is a public metabolomics database, which provides overall information of human metabolites. The HMDB contains 114,100 metabolites, 351,754 experimentally measured and computationally predicted NMR, tandem MS and GC-MS reference spectra, 25,770 illustrated metabolic pathways, and 5498 metabolite-disease associations. In addition to the large amount of data in the database, 18,192 metabolic reactions have been recently added to the HMDB [46].

The BioMagResBank (BMRB; http://www.bmrb.wisc.edu/ (accessed on 1 February 2020)) is an NMR database for the experimental and derived data collected from NMR spectroscopic studies of biological metabolites that contains four main data components [47]: Over 4500 quantitative NMR spectral parameters, databases for NMR restraints, multi-dimensional and time-domain (raw) spectral data, and over 250 metabolites with 1D- and 2D-NMR spectra.

The Kyoto Encyclopedia of Genes and Genomes (KEGG) database is one of the most popular metabolome databases, which contains information of metabolic pathways and interaction networks. The KEGG database (https://www.genome.jp/kegg/ (accessed on 1 May 2020)) combines 15 major databases [48]. The KEGG path graph uses a chart for metabolic reactions/interaction network-based species, genetic information processing, information processing environment and other cells in the process, the reaction of the human disease/interaction network, and the relationship between the drug development network (network) chemical structure transformation. In addition, it not only provides mutual biochemical substances in all possible metabolic pathways, but also includes comprehensive annotations of the enzymes that catalyze each step of the reaction [49].

The Small Molecule Pathway Database (SMPDB; http://www.smpdb.ca/ (accessed on 1 May 2020)) is an interactive, visualized data repository containing more than 30,000 small molecule pathways found in the human body. Seventy percent of these paths are unique to this database and cannot be found in other databases. Due to its availability and wide coverage, SMPDB is now integrated into several other databases, including HMDB and DrugBank.

**Figure 1:**
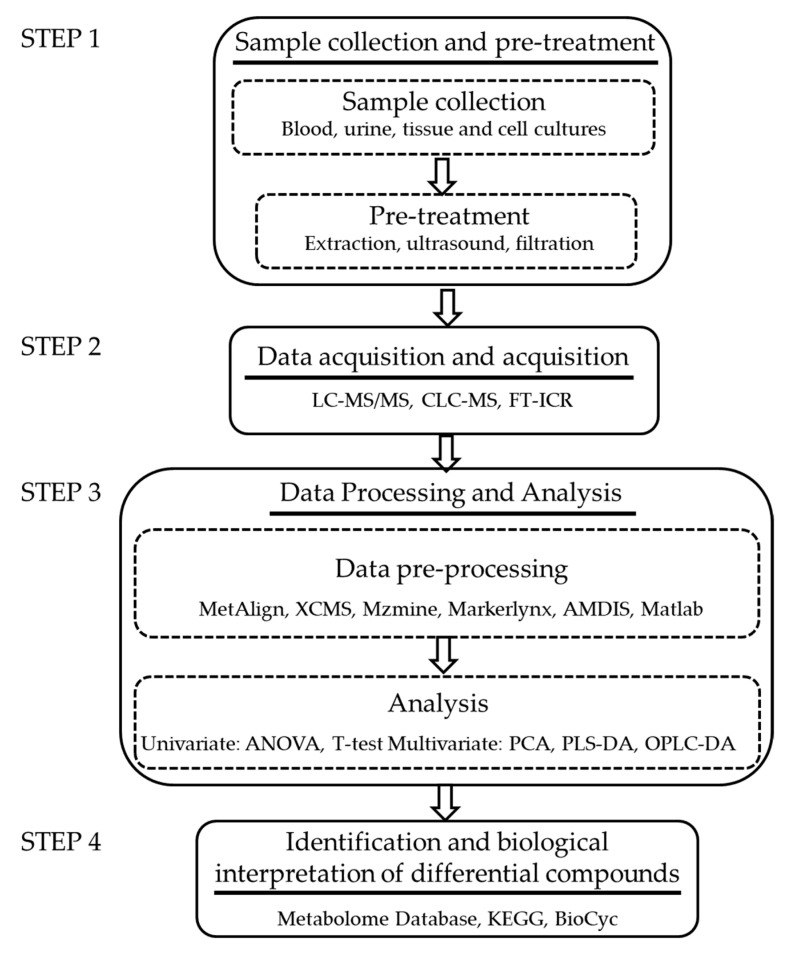
The workflow of discovering differential compounds by the LC-MS-based metabolomic method.

Here, the analysis process of finding differential compounds based on the LC-MS metabolomics method is taken as an example to illustrate how to use the database to mine the original data (Figure 1).

## 5. The Application of Metabolomics in the Interactions between Different Species

### 5.1. The Interaction Between Plants and Pathogenic Organisms

Plants are exposed to the attack of invasive microorganisms, such as fungi, invertebrates, and bacteria. During interaction with pathogens, plants can generate a series of metabolic and phenotypic changes. Plants develop different metabolic and genetic responses and activate autoimmune responses, including the synthesis of disease-resistant stimulus metabolites or the detection of a pathogen-associated molecular pattern [50,51]. Metabolomics analysis technology can be used to identify small molecules related to resistance and interactions, which provides a theoretical basis for the development of new chemical control agents [52].

**Figure 2:**
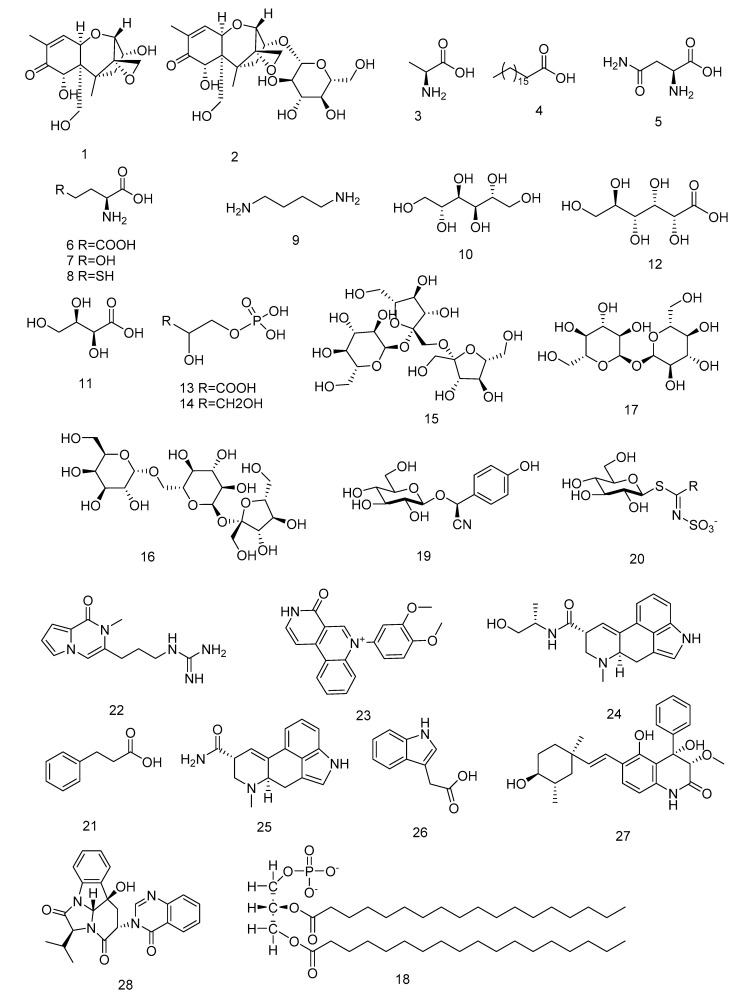
The structures of metabolites 1–28.

The genus *Fusarium* is known for its active and relatively extensive secondary metabolism involving the production of mycotoxins that are produced to kill the host, then grow and reproduce. Cajka used the LC-MS technique to describe the different metabolomic fingerprints of infected and control barley samples, and found that the mycotoxin deoxynivalenol (1) (Figure 2) and its low-toxicity glycosylated complex, deoxynivalenol-3-glucoside (2), can be used as biomarkers for screening resistant barley genotypes [53].

NMR spectroscopy and GC/LC-MS were employed to investigate the infection between a cultivated rice (*Oryza sativa*) with a blast pathogen *Magnaporthe grisea* KJ201 (compatible) and *M*. *grisea* KJ401 (incompatible). According to the metabolic profiles at each time point, there was a significant difference between KJ201- and KJ401-infected *O. sativa*, and the largest change in the metabolites was alanine (3), which was approximately 30% higher in the plants infected with the compatible strain than in the resistant plants. There was a good correlation between the time of the fungus entering the leaves and the spread of the metabolites, such as alanine. The results indicate that metabolomics has the potential for the study of biochemical changes in plant–pathogen interactions [54].

To study the interaction between *Sinorhizobium meliloti* and its host plant *Medicago sativa,* Barsch determined the metabolites for alfalfa root nodules by GC-MS, and analyzed the nodules at different stages of development to understand the metabolic changes during the formation of the nodules [55]. The metabolite levels in the nodules, roots, leaves, and flowers of symbiotic *Medicago truncatula* and *Lotus japonicas* were analyzed using GC-MS. The results showed that metabolites that were enriched in nodules included octadecanoic acid (4), asparagine (5), glutamate (6), homoserine (7), cysteine (8), putrescine (9), mannitol (10), threonic acid (11), gluconic acid (12), glyceric acid-3-P (13), and glycerol-3-P (14) [56].

In the study of host plant interactions with nematodes, Hofmann investigated the local and systemic effects of nematode infection on plant hosts using metabolomics and the gene target expression analysis [57]. They found that the levels of many amino acids and phosphorylated metabolites were increased in syncytia by the GC-MS analysis, and the high accumulation of specific sugars (such as 1-kestose (15), raffinose (16), and α, α-trehalose (17) that did not accumulate in *Arabidopsis* root controls, which indicate the potential for diagnostic and detailed metabolic analyses in future.

The interaction between *Fusarium* and wheat was studied by the UHPLC-QTOF technique [58]. The results showed that phosphatidic acid (18) is a marker of resistance to *Fusarium* infection in wheat. At the same time, with the increase in the degree of infection, the increase in oxylipin and diacylglycerol indicates that *Fusarium* invasion can activate the lipid oxidase signaling pathway.

### 5.2. Metabolomic Analyses of Plant–Herbivore Interactions

On the one hand, plants offer a nutrient-rich environment to the herbivorous insect. On the other hand, plants must reduce the damage caused by the members of other kingdoms such as nematodes and receive beneficial metabolites with a symbiotic organism. In ecology, metabolomics analysis has led to novel insights into the mechanisms of plant resistance to herbivores [59].

Plant populations are affected by a variety of biotic interactions [60] and metabolites, especially secondary metabolites, play a key role in a multitude of ecological processes, such as cyanogenic glycosides (19), glucosinolates (20), alkaloids, and terpenoids [61], which are toxic or deterrent to a wide range of herbivores. However, not all herbivores are affected by these defensive compounds. For example, small cabbage white (*Pieris rapae*) caterpillars that are specialist herbivores of wild and cultivated cabbages, possess specific P450 enzymes that disarm glucosinolates (20) before toxic products are formed in the insect’s gut with the results of detecting by GC–MS and GC–FID [62]. In turn, plants can counter the physiological adaptations of herbivores by slightly changing the molecular structure of defense compounds. Therefore, a comprehensive metabolomics analysis that includes these two compounds may be extremely useful in understanding the mechanisms of plant resistance. Riipi et al. [63] investigated the variations in the leaf chemistry of mountain birch (*Betula pubescens*), and were able to detect temporal changes in leaf metabolites, determined by HPLC, that may be used for anti-herbivory purposes, such as hydrolysable tannins and proanthocyanidins. Widarto [64] used 1D- and 2D-NMR to screen the effects of two different pests at two different instars in order to evaluate the metabolic response of rape. Using an untargeted approach, they looked at the changes in glucosinolates (20) and phenylpropionic acid (21), as well as the changes in central carbonic compounds such as sugars and amino acids.

### 5.3. Metabolomics in the Study of Symbiotic Interactions

With the rapid development of sequencing methods, it has been shown that most microbial genomes are rich in secondary metabolic synthesis gene clusters at the gene level, but they are mostly silent under normal conditions. To fully mine the bioactive components of microorganisms for drug discovery, changing the level of gene expression is one of the common ways to handle changes in culture conditions (temperature, pH, nutritional condition, etc.) and in the presence of multiple strains, the silencing of some genes is induced by microbes as a mechanism to deal with the interference of the external environment, leading to the secretion of related model molecules for defense [65]. As a means of stimulating microbial interaction, co-cultures can effectively stimulate the expression of secondary metabolites and help understand the changes in microbial interactions at the substance level.

The co-culture system of *Trametes versicolor* and *Ganoderma applanatum* is an interactive model with which to discover induced metabolites [66]. Compared to pure cultures, Yao found that 62 newly produced or significantly changed metabolites presented in the co-culture system detected by the LC-MS system and found two metabolites, identified as considerable acids. Moreover, the candidate genes of xylosyltransferase found in *T*. *versicolor* and the biological activity of the target xylose imply different response mechanisms of basidiomycete chemical defenses.

**Figure 3:**
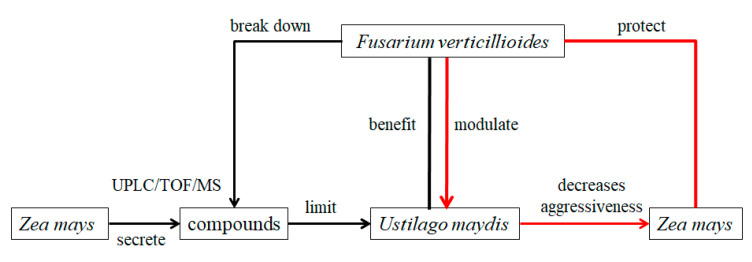
Interactions among *Fusarium verticillioides*, *Ustilago maydis*, and *Zea mays*.

The interaction between three species (the endophyte *Fusarium verticillioides*, the pathogen *Ustilago maydis*, and their shared plant host *Zea mays*) suggests that they may interact and assesses the effects of the interaction between *F*. *verticillioides* and *U*. *maydis*, both of which grow in *Z*. *mays* [66]. Thus, the endophyte *F*. *verticillioides* may function as both a defensive mutualist and a parasite, and express nutritional modes that depend on ecological context. *U*. *maydis* causes both the primary and secondary metabolisms of the host plant. This produces a dramatic change that induces the plant through the whole stage of a living nutrition defensive response. *F*. *verticillioides* modulates the growth of *U*. *maydis* and thus decreases a pathogen’s aggressiveness towards the plant (Figure 3, red), and endophytes may also break down plant compounds that limit *U*. *maydis* growth, obtaining a growth benefit from the presence of the pathogen (Figure 3, black). In the process, metabolite analyses are conducted using an UPLC/TOF/MS instrument [67].

**Figure 4:**
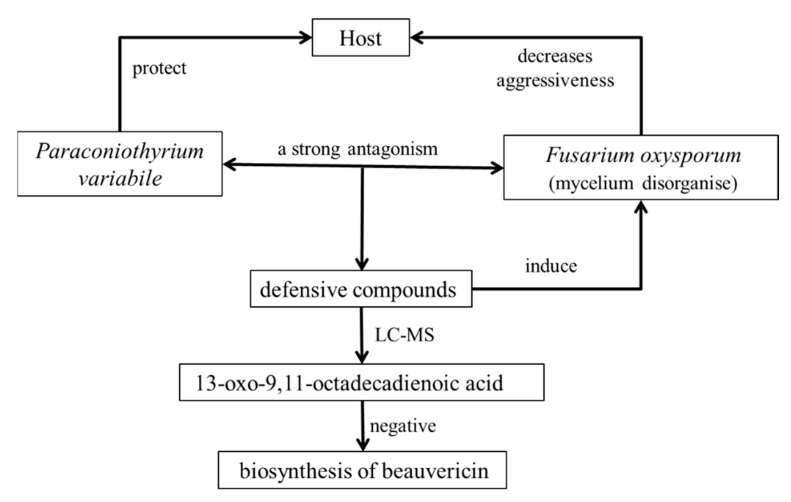
Interactions among *Paraconiothyrium variabile*, *Fusarium oxysporum*, and *Cephalotaxus harringtonia*.

*Paraconiothyrium variabile*, an endophytic fungus from the plant *Cephalotaxus harringtonia*, can inhibit the growth of common phytopathogens. Thus, *P*. *variabile* is considered a beneficial organism to a host. It has been observed that there is a strong antagonistic effect between the endophyte *P*. *variabile* and the phytopathogen *F*. *oxysporum*, and the destruction of the hyphae of *F*. *oxysporum* has been observed. More interestingly, the interactions are mediated not by the primary secondary metabolites of endophytes, but by the secondary metabolites of fungi, especially in interactions with plant phytopathogens. The metabolites induced during the competition have been analyzed by LC-MS, one of them being 13-oxo-9,11-octadienoic acid, which highlights the negative regulation of beauvericin biosynthesis as one of the most effective mycotoxins of *F*. *oxysporum* in the competition with endophytes (Figure 4). Moreover, these results suggest that certain endophytic fungi can reduce mycotoxin contamination in the control of mycotoxigenic fungi [68].

## 6. The Beneficial Interaction between Plants and Fungi

Mycorrhizal fungi are the best-studied beneficial microorganisms. Brechnmacher used GC-MS and LC-MS to analyze the metabolites of the soybean root hair with and without *Bradyrhizobium japoninum*. A total of 2910 compounds were detected, 166 of which were significantly induced by rhizobium at the early stage of infection [69]. Arbuscular mycorrhizal fungi (AMF) are soil fungi which form a mutualistic symbiosis with the roots of plants. Schweiger annotated the foliar metabolites, which are shared between species and overlap the leaf metabolic responses to AMF, to studying the symbiotic interactions between several plant species and AMF [70].

Unlike mycorrhizal fungi, which only colonize around the root, endophytic fungi can also exist in any tissues and organs.

The metabolic effects of endophytic *Neotyphodium lolii* and its host perennial ryegrass (*Lolium perenne*) in immature leaves, blades, and sheaths have been studied by direct-infusion mass spectrometry (MS). Not only have the full MS^1^ mass spectra been obtained, but also MS^2^ and MS^3^ product ion spectra, collected from the most intense MS^1^ ions. Changes in the metabolome have been detected in infected plants, with compounds such as mannitol (10), peramine (22), and perloline (23) being key compounds in infected plants [71]. Most of the endophytic fungi of ergot can improve the insect resistance of a host plant. *N. lolii*, as an endophytic fungus of *L.* *perenne*, can produce alkaloids, which have been shown to negatively affect insect pests and vertebrate herbivores. Runken horse grass (*Achnatherum inebrians*) can be toxic to many herbivores once infected by the symbiotic endophytes of *Epichloë* or *Acremonium* species due to the high ergot alkaloid concentrations in seeds and leaves identified by high-resolution FAB-MS and HPLC-FAB-MS. The contents of ergonovine (24) and lysergic acid amide (25) have been determined by HPLC at levels up to 2500 and 400 mg kg^−1^, respectively, but endophyte-free *A. inebrians* does not contain detectable levels of ergot alkaloids, which implies that *A. inebrians* can be utilized as an animal feed source if free of endophytes [72,73]. At the same time, the effects of resource supply (nitrogen and carbohydrates) on the concentrations of endophytes and endophyte-specific metabolites in the *L. perenne*–*N. lolii* association have been investigated by HPLC and qPCR, and the results indicate that the effects of endophyte infection on insect population sizes can be predicted by the concentrations of a range of metabolites other than alkaloids and depend on the insect species, fungal strain, and nitrogen supply [74].

Endophytic fungi and their metabolites can also promote the growth of plants. The production of hormone substances, such as auxin, gibberellin (GA), and cytokinin, directly promotes plant growth of host plant rice [75]. For example, with further analysis through GC-MS/SIM, *Phoma glomerata* LWL2, and *Penicillium* sp. LWL3, as well as through producing and secreting gibberellin and indole acetic acid (IAA) (26). Most of the endophytic fungi of non-ergot fungi can increase the aboveground and/or underground biomass of their hosts, which may be related to the induction and synthesis of plant hormones [76]. In addition, *Piriformospora indica* can establish mutualism with *Arabidopsis thaliana*, and promotes its growth, in a manner similar to AMF. Membrane-associated proteins were separated by 2-DE and identified by ESI-MS [77].

Endophytic fungi increase the stress and disease resistance of host plants. In nature, endophytic fungi can directly produce antagonistic substances to help plants resist pathogenic microorganisms or can indirectly induce and activate the plant defense system, so as to improve the plant defense ability and adapt to the environment of diseases and insect pests. The antimicrobial substances produced by endophytic fungi mainly include small-molecular-weight active substances and antimicrobial peptides. Luo isolated a strain of *Aspergillus racemose* from mangrove plants in Hainan, which produces the metabolites 22-epi-aflaquinolone B (27) and 14-epi-isochaetominine C (28), identified by NMR and MS spectrum data, which were used against wheat total erosion bacteria and wheat scab in agricultural disease bacteria, respectively [78].

Using UHPLC-Q-TOF/MS, Skoneczny developed an effective platform allowing for the rapid identification and accurate profiling of numerous structurally similar, difficult-to-separate bioactive isohexenylnaphthazarins (shikonins). Through experiments with two species of *Echium*, it has been identified at the physiological level that root shikonin plays an important role in the plant phenological stage at the population size [79].

## 7. Discussion and Prospects

Metabolomics is an indispensable part of systems biology research, which has made great achievements in revealing gene function, phenotypic studies, disease type, and drug toxicity evaluation. In recent years, despite the rapid development of analytical technologies, metabolomics is still in the development stage, and still faces enormous challenges in terms of technology, methods, and applications, which needs to be compatible with the study of various organisms.

In terms of metabolomics analysis technology, due to the complexity of the metabolic process of living systems and the sensitivity to changes in the surrounding environment, both measurement and sample preparation processes affect metabolomics. Thus, simple and repeatable sample preparation methods, unbiased high-sensitivity detection and analysis methods, and a wide dynamic range and flux are the key points to obtain accurate metabolomics data, but the existing methods are not widely used. In addition, the structure identification of metabolites is also a key and difficult problem in current metabolomics research. Due to the lack of a standard and universal mass spectrometry database, the application of chromatography-mass spectrometry combined technology in metabolomics research is restricted to some extent. More and more attention is being paid to the construction of a well-functioning metabolite database and the standardization of metabolomics research.

In terms of application, the survival and development of metabolomics requires not only the integration with other omics data, but also its characteristics, which can answer biological questions that other omics cannot answer from phenotypes. At present, metabolomics has been combined with other omics to analyze the experimental results of biological system changes in a comprehensive way to obtain more useful information, such as the combination of metabolomics and proteomics [80] and the combination of metabolomics and transcriptomics [81,82]. The combination of different levels of analysis, such as gene expression and regulation and protein synthesis and expression, helps clarify the biological processes that control the level of metabolites and further determine the related biomarkers, which will help comprehensively analyze molecular defense mechanisms. However, research on multi-platform comprehensive utilization systems biology is still lacking. The manner in which to combine metabolic data with data from high-throughput technologies such as genomics, transcriptomics, proteomics, metabolic pathways, and phenotypic analysis to explain the biological function and to provide a biological explanation is still far in the future. Similar to other omics, the manner in which to overcome the bottleneck and identify specific biomarkers (especially low abundance biomarkers) from a large number of metabolites is an important factor to determine whether this technique can be widely used in various fields.

Finally, metabolomics in the interaction between different species is still in the preliminary stage of development, and there are still problems to be solved in both methodological and applied studies. The main development directions in the future include: (1) The development of more broad-spectrum, in situ, real-time, universal detection methods based on the existing independent analysis platform; (2) Establishing a new metabolome analysis platform to realize the parallel analysis and data integration of multiple analysis platforms, so as to truly realize the unbiased, highly sensitive, and high-throughput analysis of metabolites in organisms; (3) Developing accurate and efficient tools for the identification or annotation of samples; (4) Further integrating metabolomics data with other omics data (genome, transcriptome, proteome, phenotype, etc.) in order to better elucidate the molecular mechanisms of biological processes and to promote their biotechnological applications.

## Figures and Tables

**Table 1:** Advantages and disadvantages of the analysis technologies for metabolomics.

Metabolomic Technology	Advantage	Disadvantage
GC-MS	High sensitivity; Easy to operate; Economical; Search the unknown material spectrum library; Suitable for volatile and non–thermosenitive compounds.	The compound may change; Not suitable for less volatile compounds.
LC-MS	Suitable for metabolites with low volatility; Poor thermal stability and mixture analysis; High sensitivity; Wide range; Fast; Qualitative and quantitative analysis.	Poor ability to distinguish isomers and stereochemistry; Poor reproducibility; Strict operating conditions; Memory effects and ion sources contamination; Complex and expensive operation; Quality discrimination effect.
ES-MS	No special treatment for samples; Low dosage; Economical; Fast; High sensitivity; High-throughput; Short test time; Multiple modes.	Poor preparation ability; Low sensitivity in some detection methods; Low separation reproducibility.
MSI	No fluorescence or radioisotope labeling; Wide mass range; High-throughput; High efficiency; Spatial resolution; Molecular specificity.	New matrix development and its application is demanded in MALDI-MSI.
NMR	Non-destructive sample; Quantified directly; High NMR signal intensity isotopic natural abundance show extraordinarily narrow line widths; Broad (~200 ppm) chemical shift rane.	Magnetic nuclei only; Low sensitivity; A greater likelihood of overlapping peaks; Low sensitivity.

## Data Availability

The data presented in this study are available in article.
